# Visualization of Peripheral Blood Vessels on the Lingual Aspect of the Mandible Using a Balanced Steady-State Free-Precession Sequence with a Time–Spatial Labeling Inversion Pulse: Usefulness for Prevention of Severe Complications of Dental Implantation

**DOI:** 10.3390/jcm11206137

**Published:** 2022-10-18

**Authors:** Tatsurou Tanaka, Yusuke Kawashima, Masafumi Oda, Nao Wakasugi-Sato, Shinobu Matsumoto-Takeda, Shun Nishimura, Yasuhiro Morimoto

**Affiliations:** 1 Department of Maxillofacial Radiology, Graduate School of Medical and Dental Sciences, Kagoshima University, Kagoshima 890-8580, Japan; 2 Division of Oral and Maxillofacial Radiology, Kyushu Dental University, Kitakyushu 803-8580, Japan

**Keywords:** blood vessels, mandibles, dental implants, steady-state free-precession, magnetic resonance angiography

## Abstract

The aim of this study was to evaluate whether a balanced steady-state free-precession (SSFP) sequence with a time–spatial labeling inversion pulse (time–SLIP) without contrast medium could elucidate branches of the lingual and facial arteries on the lingual aspect of the mandible as a potential technique for preventing severe complications in dental implantation surgery. In this study, magnetic resonance angiography (MRA) using SSFP with a time–SLIP was evaluated in 40 subjects. The outline and course of branches of the lingual and facial arteries near the mandible were assessed clinically in the same subjects against contrast-enhanced computed tomography (CT) images as the gold standard. The submental, sublingual, and deep lingual arteries could be visualized via MRA in 16, 20, and 16 of the 40 subjects, respectively. The major axes of the respective arteries were approximately 24, 24, and 16 mm. The outline and course of all visualized arteries coincided with those on CT. MRA using SSFP with a time–SLIP appears to have potential as a non-contrast technique for visualizing branches of the lingual and facial arteries on the lingual aspect of the mandible. Information regarding the outline and course of these arteries as obtained using this MRA technique could assist in preventing severe complications in dental implantation surgery.

## 1. Introduction

Dental implants can provide a better understanding of the biologic principles that direct the development of a dynamic interference between the living tissue and an artificial structure, and the technique of dental implantation to replace missing teeth can improve low occlusion power. However, severe complications can occur in dental implantation surgery [1,2]. Serious adverse events have been reported, such as death due to hemorrhage and suffocation after and/or during dental implantation in the mandible [3,4,5]. Accordingly, it is important to fully comprehend the outline and course of peripheral blood vessels on the lingual aspect of the mandible to prevent such complications. Previous studies have described mainly the foramina and grooves, whereas the descriptions of oral anatomy provided in texts are based on cadavers [6,7]. In the clinical setting, it is very difficult prior to dental implantation to examine the paths of peripheral blood vessels on the lingual side of the mandible using contrast-enhanced computed tomography (CT), in terms of the side effects of contrast media.

Alternatively, noninvasive magnetic resonance imaging (MRI) techniques, such as magnetic resonance angiography (MRA) without contrast medium, have been developed to identify and characterize peripheral blood vessels located in the thoracic, abdominal, lower extremity, head, and neck regions [8,9,10,11,12,13,14,15,16,17]. We have also conducted several studies regarding the delineation of peripheral blood vessels in the head and neck region [8,12,13]. In one of these, we reported non-contrast-enhanced MRA using steady-state free-precession (SSFP) with a time–spatial labeling inversion pulse (time–SLIP) as a highly useful technique for visualizing thin, main peripheral arteries in the oral and maxillofacial regions. This balanced SSFP technique provides a very high signal-to-noise ratio; a strong contrast between the blood vessels and tissue, such as muscle, can be obtained. Therefore, the time–SLIP technique can selectively depict arteries with good background signal suppression without using a contrast medium. Therefore, we speculated that MRA using SSFP with a time–SLIP could be used to visualize the submental, sublingual, and deep lingual arteries and elucidate their relationship to the mandible as a non-invasive examination prior to dental implantation. To the best of our knowledge, no report has investigated the course of branches of the lingual and facial arteries on the lingual side of the mandible using this MRA technique.

The aim of this study was to evaluate the ability of non-contrast-enhanced MRA using SSFP with a time–SLIP to visualize the submental, sublingual, and deep lingual arteries, and to compare its ability to elucidate their relationships to the mandible with that of contrast-enhanced CT as the gold standard.

## 2. Subjects and Methods

### 2.1. Study Subjects

The images used in the study were 40 pairs of contrast-enhanced CT scans and images from MRA using SSFP with a time–SLIP that were obtained in 40 patients with oral cancer (20 males, 20 females; mean age, 62.5 years; range, 45–85 years) who were treated at the Division of Oral and Maxillofacial Radiology in Kyushu Dental University Hospital between 2013 and 2021. MRI and contrast-enhanced CT data were used that were inspected during a similar period. Approval for the study was obtained from the institutional review board of Kyushu Dental University (Nos. 17–49, 12–21).

Contrast-enhanced CT was performed with an Activion 16 scanner (Toshiba Co. Ltd., Tokyo, Japan). To better visualize the vascular structures, each patient received 50 mL of iohexol (300 mgI/mL; Omnipaque 300™, Daiichi Pharmaceutical Co. Ltd., Tokyo, Japan) intravenously at the initiation of scanning, and an additional 50 mL by intravenous infusion during the scan. Axial scanning was performed without angulation, and 1-mm-thick, contiguous sections were obtained from the cavernous sinus to the thoracic inlet. Images were processed with standard algorithms and displayed with soft-tissue windows.

All magnetic resonance (MR) imaging was performed using a 1.5-T, full-body, MR system (Excelart Vantage™ Atlas; Toshiba, Tokyo, Japan) and a surface coil suitable for visualizing peripheral blood vessels in the oral and maxillofacial regions. Conventional, single-section sagittal, coronal, and axial scout images of the head and neck were obtained. The submental, sublingual, deep lingual, lingual, facial, and maxillary arteries on the side opposite to the oral cancer lesion were identified on an initial set of coronal T2-weighted images acquired with fat saturation to suppress signals from fat. The imaging volume for SSFP with a time–SLIP was then positioned to include both mandibles. The imaging parameters used in each sequence are shown in Table 1.

For SSFP with a time–SLIP, a peripheral-pulse-wave-gated preparation scan was performed to select the optimal delay time from a pulse-wave trigger. Several axial SSFP images with different delay times were acquired with the following parameters: repetition time (TR), 4.2 ms; echo time (TE), 2.1 ms; flip angle, 63 degrees; field of view (FOV), 225 × 300 mm; matrix, 192 × 256 pixels; slice thickness, 10 mm; slice number, 3; average acquisition time, 51 s. The resulting images were inspected visually and the delay time that best avoided signal void due to arterial flow was selected individually for each subject. Peripheral-pulse-wave-gated three-dimensional (3D) balanced SSFP images with fat saturation were then obtained in the axial plane with the following parameters: TR, 5.0 ms; TE, 2.5 ms; flip angle, 120 degrees; FOV, 300 × 300 mm; matrix, 256 × 256 pixels; slice thickness, 3.0 mm; slice number, 30; number of acquisitions, 1; parallel imaging factor, 2 in the phase-encoding direction. The raw data were zero-filled, and the posterior border of the FOV was positioned appropriately at the superior edge of the fifth cervical vertebra.

Imaging was obtained with balanced SSFP with a time–SLIP with an inversion time (TI) of 1100 ms. The acquisition time for each sequence was determined according to the subject’s pulse rate and ranged from 3 min to 5 min 30 s (mean, 4 min 10 s). A spatially selective inversion-recovery pulse with a width of 230 mm was placed above the inferior edge of the mandible to depict the inflowing arterial signal as bright blood with background signal suppression above the distal common carotid artery so as to cover the external carotid artery system.

### 2.2. Image Analysis

After acquisition, the SSFP images were post-processed to yield maximum intensity projection (MIP) reconstructions. The acquired 3D images could be reformatted into any required orientation. Two experienced radiologists (M.O. and T.T.) performed a subjective assessment of the source and the MIP MRA images using SSFP, including branches of the lingual (sublingual and deep lingual) arteries and facial (submental) artery on the lingual aspect of the mandible. The arteries on the opposite side to the oral cancer were evaluated. Visualization of the sublingual artery, deep lingual artery, submental artery, facial artery, lingual artery, or maxillary artery was scored using a 4-point scale: 0, difficult to detect even if each original image was obtained from an MRI scan; 1, detectable on the original image but difficult to detect on the reconstituted MIP image from the original images; 2, mostly detectable on the reconstituted MIP image, but some areas undetectable; 3, depiction of the entire artery with good continuation on the MIP image. If a discrepancy arose between the radiologists’ scores, consensus was reached by discussion. The kappa statistical test indicated good diagnostic agreement between the radiologists (K = 0.81).

The paths of the sublingual, deep lingual, and submental arteries were identified based on the modified method of Kami et al. [18] These arteries proceed anteriorly beneath the mylohyoid muscle and extend under the mandible into the anterior belly of the digastric muscle. The MRA was evaluated in detail to determine the location and major axis of each artery with respect to the closest tooth or equivalent part, such as mandibular 1, 2, 3, …, using contrast-enhanced CT as the gold standard. Detection rates of the sublingual, deep lingual, and submental arteries at each respective tooth on the MRA were calculated.

### 2.3. Statistical Analysis

All statistical analyses were performed using SPSS version 11 statistical software (SPSS, Chicago, IL, USA). Differences in mean values between groups were analyzed using the Mann–Whitney U test. Categorical variables were compared by a χ^2^ test. Results were considered significant for values of *p* < 0.05.

## 3. Results

### 3.1. Conspicuity in the Visualization of the Branches of Lingual and Facial Arteries around the Lingual Sides of Mandibles Using SSFP with a Time–SLIP

**Figure 1:**
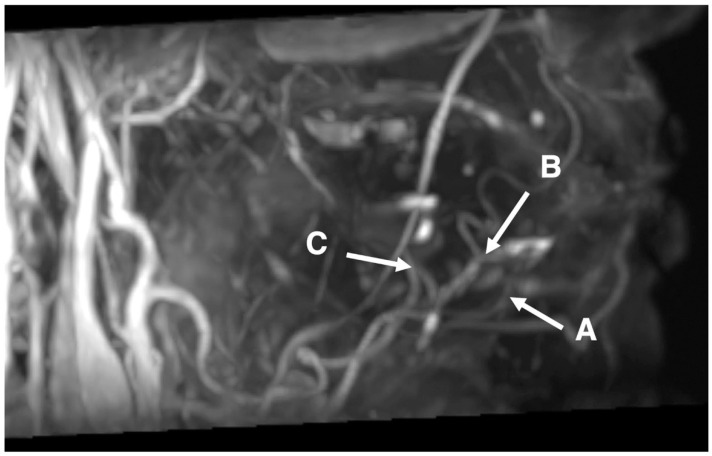
Image of 46-year-old male with left tongue carcinoma. Representative images of the submental, sublingual, and deep lingual arteries obtained by 3D MRA using SSFP with a time–SLIP. A: possible course of the submental artery around the area of the incisors; B: possible course of the sublingual artery around the incisors from premolar areas; and C: possible course of the deep lingual artery around the area of the molars.

The vasculature of branches of the lingual and facial arteries was obtained by 3D MRA using SSFP with a time–SLIP in all 40 subjects. Blood vessels were visualized at 3–5 min after initiation of scanning. Figure 1 shows representative MRA images of blood vessels around the lingual side of the right mandible that were obtained in a 46-year-old male with left tongue carcinoma. The sublingual, deep lingual, submental, facial, lingual, and maxillary arteries were more clearly visible on images obtained using SSFP with a time–SLIP (Figure 1) than on contrast-enhanced CT. The conspicuity of the submental, sublingual, deep lingual, facial, lingual, and maxillary arteries was 1.80 ± 1.22, 1.98 ± 1.19, 1.83 ± 1.17, 2.48 ± 0.88, 2.48 ± 0.75, and 2.45 ± 0.81 points, respectively (Table 2).

### 3.2. Detectable Rate and Major Axis in the Visualization of the Branches of Lingual and Facial Arteries around the Lingual Sides of Mandibles Using SSFP with a Time–SLIP

The submental artery (branch of the facial artery) was detectable in 16/40 subjects (40%), and its course was observed in the region of the incisors. The sublingual artery (branch of the lingual artery) was detectable in 20/40 subjects (50%), and its course was observed in the regions of the canines and premolars. The deep lingual artery (branch of the lingual artery) was detectable in 16/40 subjects (40%), and its course was observed in the region of the molars. In all regions, arterial branches were significantly more detectable in males than in females (χ^2^ test, *p* = 0.05) (Table 3). In the detectable arteries, the mean major axis of the submental arteries was 23.7 ± 6.3 mm (males 25.2 ± 6.3 mm, females 21.8 ± 6.0 mm), that of the sublingual arteries was 24.4 ± 9.3 mm (males 26.9 ± 9.0 mm, females 20.6 ± 8.9 mm), and that of the deep lingual arteries was 15.5 ± 7.7 mm (males 16.3 ± 9.7 mm, females 14.3 ± 2.6 mm). In all branches, the major axes were significantly thicker in males than in females (Mann–Whitney U test, *p* < 0.05) (Table 4).

### 3.3. Characteristics of Branches of Lingual and Facial Arteries Using SSFP with a Time–SLIP

In 16/40 subjects, MRA and contrast-enhanced CT showed the sublingual artery branching from the lingual artery in the molar area, running anteriorly side-by-side with the sublingual vein and distinct to the vein. In two cases, branching of the sublingual artery from the lingual artery was visualized in the molar area by both modalities, but the anterior course could be visualized only on contrast-enhanced CT.

In 20/40 subjects, MRA and CT showed the sublingual artery running together with the sublingual vein. The arteries and veins on all subjects were almost indistinguishable by MRA or CT. There was no difference in findings between MRA and CT. An irregular pattern was seen in one subject. In another subject, the lingual artery penetrated the mylohyoid muscle near the canine before running into the submandibular space to communicate with a branch of the facial vein, and then ran forward to the anterior part of the mandible.

**Figure 2:**
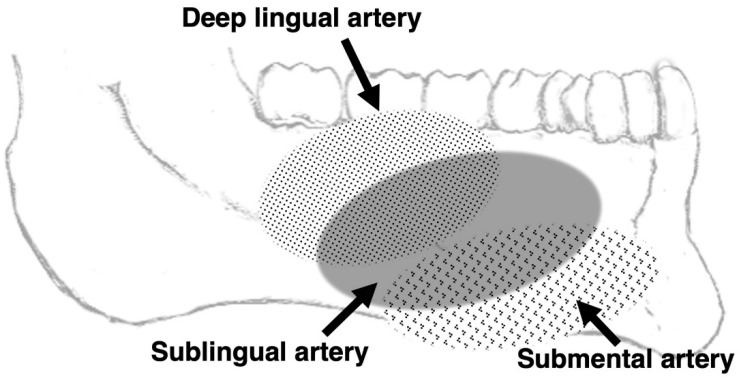
Image from 3D MRA using SSFP showing the mediodistal ranges of the visualized lingual and sublingual arteries with reference to the dentition.

Figure 2 summarizes the mesiodistal ranges of the visualized lingual arteries with reference to dentition.

## 4. Discussion

### 4.1. Applicability to Preoperative Consultation for Dental Implants

Serious adverse events during and/or after dental implantation in the mandible have been reported, including deaths from hemorrhage and suffocation. The major cause of these accidents was damage to the sublingual, deep lingual, or submental artery [1,2,3,4]. Therefore, the presence and paths of branches of the lingual and facial arteries on the lingual aspect of the mandible should be determined in detail by non-invasive examination prior to implantation. In the present study, we achieved clear and precise visualization of the sublingual, deep lingual, and submental arteries using non-contrasted 3D MRA images obtained by SSFP with a time–SLIP. The outline and course of each artery seen on MRA coincided with those seen on contrast-enhanced CT and were in agreement with the accepted anatomy (as described in an anatomy textbook and in previous reports based on autopsy) [14,18]. Therefore, we consider that MRA using SSFP with a time–SLIP has potential for clinical application for the identification of branches of the facial and lingual arteries on the lingual aspect of the mandible. In addition, detectable submental, sublingual, and deep lingual arteries all travel along the contiguous lingual side of the incisor, premolar, and molar regions of the mandible, and the detection rate on MRA using SSFP with a time–SLIP was approximately 40–50% in all subjects. The distance between each of the three arteries and the mandible was about 1–2 mm. Therefore, to prevent severe complications from dental implant treatment, we should pay attention to the course of the branches of the lingual and facial arteries along the lingual side of the mandible, which can be visualized by SSFP with time–SLIP.

### 4.2. Efficacy of MRA Technique Using SSFP with a Time–SLIP

The balanced SSFP technique provides rapid imaging with a very high signal-to-noise ratio, and intrinsic image contrast is determined by the T2/T1 ratio of the tissue. Strong contrast can be obtained between blood vessels and muscle; veins, salivary gland ducts, and arteries all show high signal intensity [14]. The time–SLIP technique enables selective depiction of the arteries with good background signal suppression, without requiring contrast medium [19]. Therefore, the combination of balanced SSFP and a time–SLIP can visualize the peripheral arterial branches of the external carotid arteries more precisely than is possible with other sequences [11,12,13]. In addition, an electrocardiogram is not required for an MRA obtained using SSFP with a time–SLIP, and mean acquisition time is around 5 min [12,16], which is tolerable for patients and short enough to avoid movement artifact. Examination of the branches of the lingual and facial arteries around the lingual aspect of the mandible using this MRA technique prior to dental implantation shows promise for preventing severe complications during surgery. In addition, SSFP with a time–SLIP is not prone to pulsation artifacts. The superior visualization of thin, main peripheral arteries on MRA obtained using this technique is due to the synergistic effects of technical superiority and shorter acquisition time. We propose that MRA examination using SSFP with a time–SLIP without contrast medium should be applied clinically for visualizing branches of lingual and facial arteries around the lingual aspect of the mandible to prevent the occurrence of severe complications in dental implantation.

### 4.3. Limitations of MRA Technique Using SSFP with a Time–SLIP

One limitation of the MRA technique using SSFP with a time–SLIP is the detection rate of approximately 40–50%, which could be improved by increasing the signal-to-noise ratio and the resolution. However, as such measures would significantly increase the scan time, which in turn adds factors such as patient pain and motion artifacts, it is necessary to consider the balance between these two needs.

Another limitation of this study is that we did not perform a direct comparison of MRA images obtained using SSFP with a time–SLIP with those obtained using contrast medium or with conventional angiography. Furthermore, no comparison was made with the ultrasonographic depiction of blood vessels. Further study is therefore needed for accurate assessment of this technique.

## 5. Conclusions

In the present study, we performed 3D MRA using the SSFP sequence with a time–SLIP, without contrast medium, to clarify whether branches of the lingual and facial arteries around the lingual aspect of the mandible could be visualized. Detailed knowledge of the course and position of these arteries could help prevent severe complications in dental implantation surgery. The submental, sublingual, and deep lingual arteries could be visualized in 16, 20, and 16 subjects, respectively, via MRA in the 40 subjects. The major axes of the respective arteries were about 24, 24, and 16 mm. The outline and course of each artery seen on MRA coincided with those seen on contrast-enhanced CT. These results suggest that MRA using SSFP with a time–SLIP is a potential non-contrast technique for visualizing the branches of the lingual and facial arteries around the lingual aspect of the mandible. Detailed examination by this technique prior to dental implantation could help prevent severe complications during and after implantation surgery.

## Figures and Tables

**Table 1:** Imaging parameters.

	Sequences		
	SSFP with a Time–SLIP	T1WI	STIR
TR (ms)	5	820	4700
TE (ms)	2.5	15	75
Flip angle (°)	120	90	90
FOV (mm)	300 × 300	250 × 225	250 × 225
Section thickness (mm)	3	6	6
Intersection gap (mm)	0	1.2	1.2
Matrix (pixels)	256 × 256	224 × 320	272 × 272

TR: repetition time, TE: echo time, FOV: field of view, SSFP: steady-state free-precession, SLIP: spatial labeling inversion pulse, T1WI: T1-weighted image, STIR: short T1 inversion recovery.

**Table 2:** Conspicuity in the visualization.

	Male (*n* = 20)	Female (*n* = 20)	Total (*n* = 40)
Submental artery	1.95 ± 1.19	1.65 ± 1.27	1.80 ± 1.22
Sublingual artery	2.20 ± 1.10	1.75 ± 1.25	1.98 ± 1.19
Deep lingual artery	2.00 ± 1.17	1.65 ± 1.18	1.83 ± 1.17
Facial artery	2.55 ± 0.89	2.40 ± 0.88	2.48 ± 0.88
Lingual artery	2.50 ± 0.76	2.45 ± 0.76	2.48 ± 0.75
Maxillary artery	2.55 ± 0.69	2.35 ± 0.93	2.45 ± 0.81

The data are expressed as a number (*n*), mean ± standard deviation.

**Table 3:** Detectable rate.

	Male (*n* = 20)	Female (*n* = 20)	Total (*n* = 40)
Submental artery	9 (45%)	7 (35%)	16 (40%)
Sublingual artery	12 (60%)	8 (40%)	20 (50%)
Deep lingual artery	10 (50%)	6 (30%)	16 (40%)

The data are expressed as a number (*n*).

**Table 4:** Major axis of detectable arteries.

	Male (*n* = 20)	Female (*n* = 20)	Total (*n* = 40)
Submental artery (mm)	25.2 ± 6.3	21.8 ± 6.0	23.7 ± 6.3
Sublingual artery (mm)	26.9 ± 9.0	20.6 ± 8.9	24.4 ± 9.3
Deep lingual artery (mm)	16.3 ± 9.7	14.3 ± 2.6	15.5 ± 7.7

The data are expressed as a number (*n*), mean ± standard deviation.

## Data Availability

Not applicable.
